# Initiating luteal phase support with sc progesterone based on low serum progesterone on the transfer day in true natural cycle frozen embryo transfers

**DOI:** 10.3389/fendo.2023.1278042

**Published:** 2023-10-23

**Authors:** Cem Demirel, Pınar Özcan, Fırat Tülek, Hikmet Tunç Timur, Özge Pasin

**Affiliations:** ^1^ Department of Obstetrics and Gynaecology, Acibadem University School of Medicine, Istanbul, Türkiye; ^2^ Department of Obstetrics and Gynaecology, Bezmialem University School of Medicine, Istanbul, Türkiye; ^3^ Department of Obstetrics and Gynecology, Dokuz Eylul University School of Medicine, Izmir, Türkiye; ^4^ Department of Biostatistics and Medical Informatics, Bezmialem University School of Medicine, Istanbul, Türkiye

**Keywords:** luteal phase support, *in-vitro* fertilisation, progesterone, frozen embryo transfer, natural cycle

## Abstract

**Introduction:**

Concerning contemporary *in-vitro* fertilisation (IVF) practice, the use of frozen embryo transfer (FET) cycles has become more common than fresh transfers. Natural cycle (NC), programmed artificial cycle and mild stimulation cycle are primary endometrium preparation cycles. Monitoring serum progesterone levels in FET cycles are in the scope of current research focus. Low progesterone levels on the day of embryo transfer is presumed to negatively affect pregnancy outcomes, while progesterone supplementation may improve pregnancy rates. The purpose of our trial is to evaluate whether initiating subcutaneous (SC) progesterone supplementation on the day of embryo transfer when serum progesterone levels are below 10 ng/mL in tNC-FET will result in pregnancy rates comparable to those of patients with sufficient serum progesterone.

**Methods:**

Retrospective single centre study was conducted between August 2022 and April 2023 with 181 tNC-FETs. Patients were separated into groups according to serum progesterone concentrations (≥10 ng/mL and <10 ng/mL) on embryo transfer (ET) day. S.c progesterone (25 mg) was given on the day of ET when serum progesterone was <10 ng/mL, continuing until the 10th gestational week. Blood samples for pregnancy tests were collected 12 days after ET. Outcome parameters were pregnancy rate, clinical pregnancy rate (CPR), miscarriage rate, multiple pregnancy rate, biochemical pregnancy, and ongoing pregnancy rate (OPR).

**Results:**

About half (49.7%) had adequate progesterone concentrations (≥10ng/mL) on ET day. There was no significant difference between the groups regarding positive pregnancy test, OPR, multiple pregnancies, and miscarriage rates (57.8% versus 52.7%; 34.4% versus 29.7%, 1.1% versus 2.2%; 7.8% versus 5.5%; respectively, for progesterone concentrations on ET day ≥10 ng/mL and <10 ng/mL). With 55.2% of transfers leading to clinical pregnancy, significant differences emerged in biochemical pregnancy and CPR (3.3% vs 12.1%, P=0.02; 54.4% vs 40.7%, P=0.03, for ≥10 ng/mL and <10 ng/mL progesterone concentrations on ET day).

**Discussion:**

This study indicates that nearly half of the tNC-FETs may need luteal phase support due to low progesterone. However, 25 mc sc progesterone rescued the luteal support and yielded similar OPR as compared to normal progesterone group. Further studies are needed for understanding optimal progesterone levels, supplementation effectiveness, and potential benefits of earlier supplementation in FETs.

## Introduction

In today’s IVF practice, frozen embryo transfer (FET) cycles have started to override fresh transfers, owing to the improvements in vitrification techniques along with the added benefits of elimination of ovarian hyperstimulation syndrome (OHSS) and improved success rates in hyper-responders when freeze-all strategy is used. Increased utilisation of preimplantation genetic screening of embryos further augments the switch to frozen transfer cycles (1, 2). According to a United States nationwide database, FET cycles have recently become approximately 77.0% of all ETs (3). There are mainly three strategies utilised, each having further modifications within, for endometrium preparation during FET, namely natural cycle, programmed artificial cycle, and mild stimulation cycle FETs. Until very recently, there had been no clear-cut consensus yet for the optimal FET strategy, but the recent trend is more towards the use of a natural cycle (4–7). The reasons are primarily two folds, one being a higher ongoing pregnancy rate compared to artificial cycles due to a lower incidence of early pregnancy loss and the other one being the pregnancy complications like pregnancy induced hypertension and significant for gestational age fetuses that are unique to artificial cycles, most probably due to the absence of corpus luteum (8). Nowadays, the main research topic is focused on monitoring FET cycles best and finding out the confounding factors that may affect success rates. Monitorisation of serum progesterone levels is a central topic of interest in FET cycles, and it has been documented in various reports that serum progesterone levels below a certain threshold around the time of ET result in decreased odds for live birth rates. This has been verified in artificial FET cycles where serum progesterone levels lower than 8-10 ng/mL on the day or preceding day of ET are shown to adversely affect the live birth rates (9, 10). Rescue strategies in the form of adding 25 mg of supplemental subcutaneous (SC) progesterone are shown to restore live birth rates to those of patients with adequate serum progesterone levels in artificial FET cycles, to overcome the deleterious effect of the insufficient progesterone support of the endometrium (11, 12).

The corpus luteum (CL) is involved in a natural cycle FET. Nevertheless, progesterone production by CL in some cycles may be insufficient to provide adequate support for implantation and continuation of pregnancy. A very recent meta-analysis of randomised controlled trials for luteal phase support (LPS) in natural cycle FET (NC-FET) has shown that progesterone supplementation for LPS was associated with increased LBR and CPR in NC-FET cycles, giving additional evidence for the insufficiency of corpus luteum in some of the natural cycles (13). In the same meta-analysis, LPS improves LBR only in true NC-FET but not in modified NC-FET, where hCG used to trigger dominant follicles must act as LPS per se. A recent retrospective study has shown that patients with low serum progesterone (< 10 ng/mL) on the day before NC-FET have reduced live birth rates compared to those who have > 10 ng/mL progesterone (14). Of note, these NC-FET cases were not using any LPS, solely depending on progesterone production by CL. Since lower progesterone concentrations on ET day during natural cycles FET may negatively influence pregnancy outcomes, progesterone supplementation for the restoration of serum progesterone to adequate concentrations may be an opportunity to improve PRs (9, 14–16).

The purpose of our trial is to evaluate whether initiating SC progesterone supplementation on the day of embryo transfer when serum progesterone levels are below 10 ng/mL in tNC-FET will result in pregnancy rates comparable to those of patients with sufficient serum progesterone. In brief, to our knowledge, this is the first study to evaluate the feasibility of a rescue progesterone supplementation strategy in tNC-FET cases.

## Materials and methods

This retrospective cohort study in a single centre was carried out at the IVF centre of Acibadem Ataşehir Hospital between August 2022 and April 2023. A total of 181 true natural cycles vitrified–warmed FETs (tNC-FET) were evaluated. The Ethical Committee of Bezmialem University approved the protocol of our study (Approval no. 2023/165). Exclusion criteria were patients with cycle cancellation due to lack of a viable embryo or who had Mullerian anomalies not corrected by surgery, such as bicornuate, unicornuate, or didelphic uterus, those with a history of recurrent miscarriage, and those with a presence of hydrosalpinx. Those who had corrected uterine anomalies such as uterine septum, submucosal fibroids, or endometrial polyps were not excluded.

The collected data consisted of demographic characteristics of the patients, including age at oocyte pick up (year), IVF indications (tubal, male, unexplained, diminishing ovarian reserve, ovulatory, mixed), serum LH concentration (IU/L) on the day of LH surge, serum progesterone concentration (ng/mL) on the day of LH surge and the day of embryo transfer, endometrial thickness (mm) on the day of embryo transfer, stage of transferred embryo (Day 3/blastocyst) and number of embryos transferred. Outcome parameters analysed are pregnancy rate, clinical pregnancy rate (CPR) (fetal heartbeat by transvaginal ultrasound), miscarriage rate (any clinical pregnancy lost before the 12^th^ gestational week), multiple pregnancy rate, biochemical pregnancy, and ongoing pregnancy rate (OPR) (pregnancies beyond 12 weeks of pregnancy).

All women underwent a transvaginal ultrasound scan on the second or third day of menstruation to confirm the absence of any ovarian cyst or CL. The second control was scheduled on the eighth day of the cycle to evaluate the emergence of the dominant follicle by transvaginal ultrasonography. Endocrine monitoring with serum LH and progesterone measurements was initiated everyday at 9 am once the leading follicle attained a mean diameter of approximately 15 mm. An increase of at least 180% compared to the previous serum LH level was taken as consistent with LH surge. FETs were performed four days after the LH rise for day three embryos and six days after the blastocyst stage. Blood samples for serum progesterone concentrations were collected on ET day at 9 am for all patients. 25 mg of SC progesterone (Prolutex; IBSA, Switzerland) per day was initiated at 11 am in patients with serum progesterone concentrations <10 ng/mL on ET day. The blood samples for pregnancy tests were collected 12 days after ET. Progesterone supplementation was discontinued if there was no pregnancy. We hypothesized that patients with serum progesterone concentrations <10 ng/mL in tNC-FET on the day of embryo transfer may have insufficiency of corpus luteum. We would like to continue SC progesterone until the luteo-placental shift (10th weeks of pregnancy). Therefore, progesterone supplementation at the same dose was continued until the 10th gestational week for viable pregnancies. Serum analysis and hormone measurement, vitrification and warming procedure, and ET were performed as described in our previous study (17).

The data were analysed using SPSS Statistics for Windows, Version 26 (IBM Corp, Armonk, NY, USA). Data were reported as mean ± SD or number and percentage. The Pearson chi-squared test, Fisher’s exact and Fisher Freeman Halton tests were used to compare categorical variables. The Kolmogorov–Smirnov test was used to test the normal distribution of continuous variables. The homogeneity of variance was evaluated with Levene’s test. Student’s t-test was used to compare two independent groups regarding the means of normally distributed variables. The Mann–Whitney U-test was used to compare two independent groupsregardingf the means of non-normally distributed variables. P<0.05 was considered significant. As this is a retrospective study, no power analysis was performed prior to the study, and we included all women who underwent natural cycles FET during the period.

## Results

A total of 181 tNC- FET were analysed. The mean age was 35.37 ± 4.72 in patients with serum progesterone concentrations ≥10 ng/mL on the day of ET and 35.35 ± 5.3 years in patients with serum progesterone concentrations <10 ng/ml on ET day. Overall, 49.7% (90/181) of patients had adequate serum progesterone concentrations on ET day (≥10 ng/mL). Patients with serum progesterone concentrations <10 ng/mL and ≥10 ng/mL were similar in terms of age at oocyte pick up, IVF indications, serum LH concentration on the day of LH surge, serum progesterone concentration on the day of LH surge, endometrial thickness on the day of embryo transfer and number of embryos transferred. (**Table 1**). There was a significant difference between groups regarding serum progesterone concentrations on ET day (14.63 ± 5.57 vs 7.29 ± 1.93 ng/mL; P < 0.001, respectively, for ≥10 ng/mL and <10 ng/mL serum progesterone concentration). There was a statistically significant difference in terms of the distribution of day three embryos and blastocysts in patients with serum progesterone concentrations ≥10 ng/mL and patients with <10 ng/mL on the day of ET. The number of day three embryo transfers was significantly higher in patients with serum progesterone concentrations <10 ng/mL on the day of ET (5.6% versus 18.7%; P =0.01). There was a statistically significant difference in serum progesterone concentrations between those undergoing day 3 and day 5 frozen thawed embryo transfers (7.99 ± 6.86 vs 11.34 ± 5.23 ng/mL; P<0.001, respectively, for day three and blastocyst stages). However, there was no significant difference between the patients in terms of clinical pregnancy rates according to the day of embryo transfer (2% versus 8.1%; P=0.31).

**Table 1:** The characteristics of patients.

Variables	Progesterone ≥10 ng/mL (n=90)	Progesterone <10 ng/mL (n=91)	P value
Age at oocyte pick up (years)	35.37 ± 4.72	35.35 ± 5.3	0.98
Indication of IVF (n. (%)) Tubal Male Unexplained Ovulatory Mixed	21.1 (19) 1.1 (1) 2.2 (2) 1.1 (1) 74.4 (67)	24.2 (22) 4.4 (4) 4.4 (4) 3.3 (3) 63.7 (58)	0.22
Serum LH (IU/L) concentration at the day of LH surge	19.57 ± 9.88	19.22 ± 7.55	0.78
Serum progesterone concentration (ng/mL) at the day of LH surge	2.33 ± 10.6	1.34 ± 6.11	0.18
Serum progesterone concentration (ng/mL) on embryo transfer day	14.63 ± 5.57	7.29 ± 1.93	<0.001*
Endometrial thickness (mm) on embryo transfer day	9.05 ± 1.6	9.29 ± 1.54	0.19
Number of embryos transferred (n. (%)) 1 2	65 (72.2%) 25 (27.8%)	60 (65.9%) 31 (34.1%)	0.42
Embryo stage at transfer Day 3 blastocyst	5.6 (5) 94.4 (85)	18.7 (17) 81.3 (74)	0.01*

Values are expressed as mean± standard deviation for continuous variables and (n. (%)) for categorical variables. ^*^P<0.05. significant difference.

Of all transfers, 55.2% (100/181) of all transfers resulted in a clinical pregnancy. There was no significant difference between the groups regarding positive pregnancy test, OPR, multiple pregnancies, and miscarriage rates (57.8% versus 52.7%; 34.4% versus 29.7%, 1.1% versus 2.2%; 7.8% versus 5.5%; respectively, for progesterone concentrations on ET day ≥10 ng/mL and <10 ng/mL). Biochemical pregnancy rates (3.3% versus 12.1%, P = 0.02) was lower and clinical pregnancy rates (54.4% versus 40.7%, P = 0.03) was higher in patients with ≥10 ng/mL P concentrations on the day of ET. Serum progesterone concentrations on ET day were evaluated by percentiles (<10%, 10–49%, 50–90%, and >90%). The threshold for <10%, 10– 49%, 50–90%, and >90% were 0–5.9 (11%), 5,9–9,96 (39.2%), 9.96–16.54 (39.8%) and >16.64 ng/mL (9.9%), respectively. However, the positive pregnancy test of serum progesterone percentiles on ET day was 8% in the <10% percentile, 40% in the 10–49% percentile, 39% in the 50–90% percentile, and 13% in the >90% percentile. The CPR of serum progesterone percentiles on ET day was 5.8% in the <10% percentile, 37.2% in the 10–49% percentile, 43% in the 50–90% percentile, and 14% in the >90% percentile. There was no significant difference between the patients in terms of CPR in all percentiles when evaluating separately according to the day of embryo transfer. The CPR of serum progesterone percentiles for day three embrio on ET day was 35.3% in the <10% percentile, 41.2% in the 10–49% percentile, 17.6% in the 50–90% percentile, and 5.9% in the >90% percentile (P=0.41). The CPR of serum progesterone percentiles for blastocyst stages on ET day was 9.4% in the <10% percentile, 37.5% in the 10–49% percentile, 46.9% in the 50–90% percentile, and 6.3% in the >90% percentile (P=0.14). 10 ng/mL was represented as the threshold of the 51st percentile in this study. A total of 78.5% of biochemical pregnancies were under the 50 percentile.

## Discussion

The correlation between low serum progesterone levels around the timing of embryo transfer and decreased LBR has been demonstrated in artificial and natural frozen embryo transfer cycles (9, 10, 14). The necessity of exogenous luteal phase support has been debated in NC-FET cycles since there is a corpus luteum to produce endogenous progesterone. However, the adequacy of CL function is not absolute, and it has been shown in a recent study that low serum progesterone (<10 ng/mL) on the day before NC-FET has been associated with reduced live birth rates compared to cycles with ≥ 10 ng/mL progesterone (14). A recent meta-analysis to evaluate whether LPS is beneficial in NC-FET also supports the findings of this study, giving additional evidence for the insufficiency of the corpus luteum in some of the natural cycles. This meta-analysis showed that progesterone supplementation for LPS was associated with increased LBR and CPR in NC-FET cycles (13). Stavridis et al., have recently presented another meta-analysis. The results of this meta-analysis suggested that rescue progesterone in patients with lower serum progesterone levels results in similar CPR, OPR and LBRs to patients with sufficient progesterone levels in artificial FET (18).

The lower limit of serum progesterone concentration indicative of an adequate luteal phase in natural cycles is not yet clear. However, mid-luteal serum concentrations of about 10 ng/mL are mostly accepted as adequate progesterone production by the corpus luteum during a natural cycle (14, 19, 20). Therefore, we took 10 ng/mL as the threshold serum progesterone concentration on the ET day in our study. In our study, only 49.7% of patients who are undergoing an NC-FET reached the cutoff serum progesterone concentration (≥10 ng/mL) on ET day, but 50.3% of patients fell short of this range (<10 ng/mL). Therefore, nearly half the NC-FET cycles might have benefitted from some LPS. LPS might be routinely implemented one day after LH surge in a standard fashion or reserved for cases with low levels of serum progesterone around the time of embryo transfer/implantation. Meanwhile, the number of day three embryo transfers was significantly higher in patients with serum progesterone concentrations <10 ng/mL on the day of ET and serum progesterone concentrations on ET day were significantly lower in day three embryo transfers. Therefore, 10 ng/mL as the threshold concentration of serum progesterone on the day of ET may not be suitable for day three embryo transfers. The importance of endocrine monitoring in t-NC is still a matter of debate. A retrospective study including 610 patients underwent t-NC FET found a 28.4% incidence of serum P4 elevation before the LH surge but there was no significant difference in terms of OPR between patients with or without P4 elevation on the day of LH surge (32.5% vs 31.7%) (21). The results of subgroup analysis demonstrated that, not the level, but the duration of P4 exposure before the LH surge was associated with the lower pregnancy rates.

Our study is the first study in patients undergoing t-NC FET to evaluate the feasibility of the alternative approach, reserving the initiation of progesterone support when the serum levels of progesterone are below 10 ng/mL on the day of ET. If this approach had been practical, it might have rescued nearly half of the tNC-FET cycles with adequate serum progesterone concentrations from unnecessary progesterone supplementation.

The major finding of our study is that rescue protocol by adding SC progesterone is effective and as a result no significant difference could be shown in terms of OPR. At the same time, CPR in patients with low serum progesterone concentrations on ET day was lower despite LPS with sc progesterone supplementation. Our study has found that although pregnancy rates are similar, CPR is inferior in cycles with low serum progesterone despite the initiation of SC progesterone on ET day because of a higher incidence of biochemical pregnancies in this group. Therefore, SC progesterone supplementation can not sustain the essential luteal phase support when initiated on the day of ET if the endogenous CL function is inadequate. There are two possible explanations for this finding: Either we are too late to initiate luteal phase rescue, or the support dose/mode (sc) is inadequate. According to our best knowledge, our study is the first study to evaluate the effect of sc progesterone supplementation on PRs in tNC-FET based on serum progesterone level on ET day. Our previous study in programmed artificial FET cycles evaluated the effect of additional rescue. SC progesterone supplementation for restoring serum progesterone level in patients with low serum progesterone concentration on ET day, two days after the rescue treatment (17). However, in current study, we did not evaluate whether adequate serum progesterone level was reached with SC progesterone supplementation or not, due to its retrospective nature.

Bjuresten et al. reported an improvement in LBRs with 400 mg twice daily of vaginal progesterone in NC-FETs in their RCT, including 435 women (30% vs. 20%, P = 0.02), compared to those without supplementation (16). Likewise, in a prospective randomised controlled trial, Wanggren et al. evaluated the effect of LPS with 100 mg twice daily vaginal progesterone tablet in NC-FET cycles. They demonstrated the beneficial effect of vaginal progesterone supplementation on PRs (OR: 1.465, 95% CI 1.012–2.108, P=0.049), CPRs (OR: 1.497, 95% CI 1.024–2.188, P=0.043) and LBRs (OR:1.635, 95% CI 1.102–2.428, P=0.017) (22). In a retrospective cohort study including 228 consecutive patients who underwent NC-FET, Kim et al. reported improved LBR and reduced miscarriage rate with vaginal progesterone gel supplementation (23). Moreover, two recent meta-analyses strongly demonstrated the association between LPS with vaginal progesterone in NC-FET and higher LBRs (24, 25). In fact, according to our results, individualised rescue protocol using SC progesteron is effective since OPR is similar between groups. The primary difference was that we used an individualised LPS with SC progesterone by using serum progesterone levels on the day of ET. The optimal dosage of sc progesterone supplementation as LPS in tNC-FET has not been thoroughly studied, and further studies are needed on this aspect. Furthermore, in all those studies mentioned, vaginal progesterone as LPS was initiated before the transfer day, a RCT comparing rescue LPS with SC progesterone with no support is necessary to find out the exact role of this protocol.

We have previously mentioned a debate in the literature related to the beneficial effect of progesterone supplementation as LPS in tNC-FET. For tNC-FETs, it remains an open question about how to support the luteal phase, which dosages and agents to use, and the LPS’s efficacy for these selected agents in clinical practice. In this regard, our study may shed more light on our knowledge to affect the current practice for NC-FET.

There are few points related to the current study as limitations. The first limitation is that it is a retrospective study with a relatively small sample size and that we did not report LBRs. We do not think patients with low serum progesterone concentrations on ET day can be refrained from progesterone supplementation because of the well-documented strong relationship between adequate serum progesterone concentrations and successful implantation and pregnancy outcome. Furthermore, the design of our practice to add LPS rescue with SC 25 mg progesterone is a clinically proven mode of action in artificial FET cycles when serum progesterone levels are found in a suboptimal range for a successful outcome on the day of ET. The only difference was that this strategy was never validated in tNC-FET cases. We were expecting the same beneficial effect of this strategy in tNC-FET cases since we have an additional functioning CL in a natural cycle. The second limitation is that serum progesterone concentrations were not measured after starting progesterone supplementation to confirm the attainment of adequate levels with sc progesterone. The design of our study, SC progesterone supplementation as LPS in natural cycles FET based on serum progesterone level on ET day, might be considered as the strength of our study.

In conclusion, according to our results, almost half of the patients who undergo tNC-FET might need LPS because of low serum progesterone concentrations on ET day. The measurement of serum progesterone level on ET day may create the opportunity for progesterone supplementation for patients with low serum progesterone concentrations. On the other hand, daily 25 mg sc progesterone supplementation may achieve similar OPR. Starting luteal phase support earlier than the day of embryo transfer in all NC-FET cases may be a better clinical decision in this setting until cut-off levels of P on the day of ET in tNC-FET is established and the benefit of rescue protocol is proven by RCTs. Further well-designed randomised controlled trials with large samples of patients are needed to evaluate the cutoff level of serum progesterone, which determine the necessity of LPS and the effect of sc progesterone supplementation as LPS on LBR during NC-FET.

## Data availability statement

The original contributions presented in the study are included in the article/supplementary material, further inquiries can be directed to the corresponding author/s.

## Ethics statement

The studies involving humans were approved by The Ethical Committee of Bezmialem University. The studies were conducted in accordance with the local legislation and institutional requirements. The participants provided their written informed consent to participate in this study.

## Author contributions

CD: Conceptualization, Methodology, Writing – review & editing. PÖ: Conceptualization, Formal Analysis, Methodology, Supervision, Writing – original draft. FT: Data curation, Formal Analysis, Methodology, Writing – original draft. HT: Data curation, Formal Analysis, Writing – review & editing. ÖP: Data curation, Formal Analysis, Writing – review & editing.
